# Opiate-Related Testicular Stem Cell Niche Toxic Pathology

**DOI:** 10.5812/ijhrba.6522

**Published:** 2012-07-25

**Authors:** Albert Stuart Reece

**Affiliations:** 1 School of Psychiatry and Clinical Neurosciences, University of Western Australia, Crawley, Australia

**Keywords:** Morphine, Opioid, Testis

*Dear Editor*

The recent paper on the methadone- and buprenorphine- related testicular toxicity (1) adds a detailed study to the gonadal toxicity of opiates given over the brief period of 15 days. The study mentions the divergent published views relating to opiate-induced hypogonadism, and rightly cites the importance of hypothalamic hypogonadism as the major background endocrine factor. The real importance of this study however lies in the place it occupies in our biological understanding of the testis as an important and well described stem cell niche environment, and the place of the gonads in the panorama of opiate related organ-specific toxico pathological mechanisms. This latter effect was described by Web Appendix 6 of a report on the largest Australian opiate maintenance program with over 21 years of follow-up, documenting greatly elevated standardized mortality rates amongst decadents in the psycho neurological, pulmonary, hepatic, gastrointestinal and cardiovascular systems, and cancer- related disease (2), which strongly suggests acceleration by opiates of age-related degenerative processes. The wide variety of organs manifesting toxic pathological deleterious change strongly suggests common cellular mechanism operating in diverse tissues. Opiate related cellular dysfunction has for some time now been characterized as reduced stem cell proliferation in the setting of increased cellular loss by apoptosis and other cell death pathways compounded by dysfunctional immune stimulation which includes immunosuppression. Clearly the reduced stem cell activity is compounded by increased rates of cell death and cell senescence. In that stem cells are known to be very sensitive to immune activity, immune activation is likely to further potentiate the unfavourable cell death - cell renewal imbalance. More recent studies have shown that this also is an oversimplification. The vascular toxicity of opiates has been shown in both preclinical (3) and clinical studies. P38 Mitogen Activated Protein (MAP) kinase, toll-like receptors 2,4 and 9, c-Fos and nitric oxide synthase activation have been shown to be linked to cell death mechanisms (4), and cell membrane immune signalling has been shown to be uncoupled from nuclear factor kappa B (NF-κB) transduction. Intracellular immune receptors, Nucleotide Oligomerization Domain (NOD) 2 and Nod-Like Receptor Pyrin Containing Domain -3 (NALP3) have also been shown to be linked with cell death pathways, as have Fas-associated death domain (FADD) and Tumour Necrosis Factor (TNF) - Death receptors. Moreover opiates directly ligate the peri nuclear opiate related growth factor receptor which directly inhibits stem cell cycling by both P16 and P21 (5). Genome wide association studies suggest that the senescence and P16-locus on chromosome 9p21.3 is of pivotal importance in degenerative cardiovascular and diabetic diseases. Transforming Growth Factor-β (TGFβ) and micro- Ribo Nucleic Acid (micro-RNA) are also involved. As many of these are oncogenic pathways, this also explains the higher rate of cancer observed in several careful Iranian studies (6).

The study is also consistent with the known interdependence of the germinal centres of the testis with the surrounding Sertoli cells, and the interdependence of the cellular elements with the basement membrane and vasculature. Therefore this highly provocative careful histolopathological study invites further careful mechanistic investigations. One also notes many reports of buprenorphine abuse. A comment from the authors on the very differing doses used in their rats from those usually employed clinically, would have been of interest.
